# The effect of endometrial injury on first cycle IVF/ICSI outcome: A randomized controlled trial

**Published:** 2016-03

**Authors:** Ahmad Mahran, Mahmoud Ibrahim, Haitham Bahaa

**Affiliations:** *Department of Obstetrics and Gynecology, Faculty of Medicine, Minia University, Minia, Egypt.*

**Keywords:** *Endometrial injury*, *Implantation rate*, *Live birth rate*, *In vitro fertilization (IVF)*, *Intra cytoplasmic sperm injection (ICSI)*

## Abstract

**Background:**

Implantation remains a limiting step in IVF/ICSI. Endometrial injury isa promising procedure aiming at improving the implantation and pregnancy rates after IVF/ICSI.

**Objective:**

The aim of this study was to evaluate the effect of endometrial injury induced in precedingcycle on IVF/ICSI outcome.

**Materials and Methods:**

Four hundred patients undergoing their first IVF/ICSI cycle in two IVF units in Minia, Egypt were randomly selected to undergo either endometrial injury in luteal phase of preceding cycle (intervention group) or no treatment (control group). Primary outcome wasthe implantation and live birth ratesWhile the secondary outcome was clinical pregnancy, miscarriage, multiple pregnancy rates, pain and bleeding during and after procedure.

**Results:**

Implantation and live birth rates were significantly higher in intervention compared with control group (22.4% vs. 18.7%, p=0.02 and 67% vs. 28%, p=0.03), respectively. There was also a significant reduction in miscarriage rate in intervention group (4.8% vs. 19.7%, respectively, p<0.001).

**Conclusion:**

Endometrial injury in preceding cycle improves the implantation rate and live birth rate and reduces the miscarriage rate per clinical pregnancy in patients undergoing their first IVF/ICSI cycle.

## Introduction

Implantation remains a limiting step in IVF/ICSI. Many procedures have been tried to improve the implantation rate in IVF/ICSI cycles. Endometrial injury is one of these procedures which gained popularity in last few years. The underlying mechanism through which endometrial injury improves implantation is still unclear. There are three main supposed theories. First is through inducing decidualization of endometrium, which might improve the transferred embryos implantation (1). Second is that the process of healing after endometrial injury involves an inflammatory reaction mediated with cytokines, interleukins, growth factors, macrophages and dendritic cells, which are beneficial to embryo implantation (1-3). 

The third is that endometrial injury in previous cycle leads to better synchronicity between endometrium and transferred embryos through retarding endometrial maturation (1). Most of previously conducted studies focused on cases with repeated implantation failure (RIF). 

In this trial, the aim was to assess the effectiveness and safety of endometrial injury in previous cycle in patients with first IVF/ICSI cycle.

## Materials and methods

This study was a prospective randomized controlled trial involving four hundred patients undergoing IVF/ICSI at 2 IVF units (Minia Infertility Research Unit and Minia IVF Unit, Minia, Egypt) in the period from June 2012 to September 2014. The study was approved by the Ethical Committee of the Faculty of Medicine, Minia University, Egypt. All the patients provided written informed consent before inclusion in the study.

Inclusion criteria were: a) age between 20-40 years. b) FSH ≤12 c) normal uterine cavity by hysteroscopy (done routinely for all cases prior to ICSI) d) ≥2 good quality embryos replaced. The exclusion criteria were : a) age >40 years b) abnormal uterine cavity due to submucous fibroid, endometrial polyps, Asherman’s syndrome or congenital uterine malformations c) patients who had <2 good quality embryos at the time of transfer from analysis.

**Sample size calculation**

Sample size was calculated to prevent type II error. Live birth rate at units in which the study was conducted was 30%. To be of clinicall significance, it was assumed that endometrial injury prior to IVF/ICSI should achieve a live birth rate of 50%. Based on these data, we would need to study 91 patients in each arm to be able to reject the null hypothesis that the rates for study and control groups are equal in impalnataion and pregnancy rates with a probability of 80%. The type one error probability associated with this test for the null hypothesis is 0.05. To compensate for discontinuation, we recruited 220 patients in each arm.

**Randomization**

The study was explained to all eligible patients and they were offered to take part in study and given a patient information sheet. They were given enough time to think about. In day for hysteroscopy those who were accepted to take part in the study gave an informed consent form. Randomization was done simply using sealed envelopes before undergoing hysteroscopy.

**Hysteroscopic evaluation of the uterine cavity**

Hysteroscopy was done in all cases as per unit protocol in cycle preceding ICSI cycle. The hysteroscope used in study was a rigid continuous flow panoramic hysteroscope with 30^o^ fiberoptic lens. Hysteroscopy was performed 2-5 days postmenstrual and without anaesthesia.

**Endometrial sampling**

In intervention group, endometrial sampling was done once between 21^st^-24^th^ days of non-transfer cycle at outpatient clinic. Endometrial sampling was done using Pipelle endosampler catheter (MedGyn Endosampler, MedGyn products, Inc. USA). Scratching of fundus and posterior wall of uterine cavity was done three times. No antibiotics were prescribed to patients after the procedure.

**Treatment protocol**

Individualized protocols for both down-regulation and controlled ovarian stimulation was used. GnRH agonist midluteal protocol was used for patients ≤35 yrs, FSH <10, and antral follicle count (AFC) ≥10. Short flare protocol, or antagonist protocol was used for patients >35 yrs, FSH ≥10, and AFC <10. In long agonist protocol, patients were down regulated with 0.1 mg Decapeptyl given by subcutaneous (sc) injection (Decapeptyl, Ferring, Germany). 

The dose was then reduced to 0.05 mg and continued till HCG day. In short protocol, GnRH agonist was started on day 2 of stimulation cycle and continued till the HCG day. In antagonist protocol, GnRH antagonist was given when a leading follicle reached 14 mm and continued till day of HCG. Gonadotropin stimulation was started on day 2 or 3 of cycle after confirming pituitary desensitization. Starting dose of gonadotropins was chosen on basis of age, BMI, AFC and prior response to gonadotropin stimulation as per unit protocol. Exogenous gonadotrophins used were in form of human menopausal gonadotropins (HMG). Preparations used were Merional (IBSA, Switzerland), Menogon (Ferring, Germany) or Fostimone (IBSA, Switzerland).

Ovarian follicular responses were monitored with transvaginal ultrasound. Ultrasound scanning was started on day 8 of stimulation then every other day. Step up or step down protocols was decided according to individual patients' responses. HCG injection was given (Choriomone 10,000 IU im, Choriomone, IBSA, Switzerland) when at least 3 follicles greater than 16 mm in diameter were detected on transvaginal ultrasound scan with leading follicle reached 18-20 mm in diameter. Oocyte retrieval was performed under anaesthesia 36 hours after HCG administration. Insemination was performed by standard IVF or ICSI (IVF 143 in intervention group and 147 in control group, ICSI 57 in intervention group and 53 in the control group)

Embryos were classified according to Veeck’s grading (4) as follows: 

Grade 1: pre-embryos with blastomeres of equal size and no cytoplasmic fragmentations;

Grade 2; pre-embryos with blastomeres of equal size with cytoplasmic fragmentations equal to 15% of the total embryo volume;

Grade 3: uneven blastomeres with no fragmentations;

Grade 4: uneven blastomeres with gross fragmentation (≥20% fragments).

Cleavage-stage embryo transfer (ET) was performed on day 2 or 3. ET was performed under abdominal ultrasound guide for proper embryo placement to mid-uterine cavity. Two to five grade 1 or 2 embryos were transferred as per unit protocol. Embryo transfer was performed with Wallace catheter (Smith Medical International Ltd, Hythe, Kent, UK). Progesterone support of luteal phase was commenced on day of ET with 800 mg micronized progesterone vaginally till 12 weeks of pregnancy. Serum HCG pregnancy test was performed 14 days after ET. Ongoing pregnancies were confirmed by at least one ultrasonographically confirmed viable foetus within the uterus 4 weeks after ET.

**Outcome measures**

The Primary outcome measures of the study were:

-Live birth rate; calculated as the ratio of the number of patients with live births divided by the number of patients who had ET.

-Implantation rate; calculated as number of gestational sacs evident by ultrasound divided by the number of transferred embryos (5).

The secondary outcome measures of the study were;

-Clinical pregnancy rate; calculated as the number of the patients with clinical pregnancy (detection of fetal heart beat with ultrasound scan) divided by the number of patients who had ET.

-Miscarriage rate per clinical pregnancy; calculated as the number of patients who had a miscarriage (<24 wks) divided by the number of patients who had clinical pregnancies.

-Multiple gestations rate; calculated as the number of patients who had multiple gestation divided by the number of patients who had clinical pregnancies.

-Pain; reported pain during the intervention 

-Bleeding; reports of abnormal bleeding during or after the intervention.

**Statistical analysis**

Statistical analysis was performed using Statistical Package for Social Science (SPSS Inc, Chicago) version 17 for Microsoft Windows. Data were described in terms of mean±SEM (standard error of the mean) for continuous variables and frequencies (number of cases) and percentages for categorical data. Independent Student‘s t-test was used to compare quantitative variables and Chi square test was used to compare categorical data. P<0.05 was considered significant.

## Results

**Figure 1:**
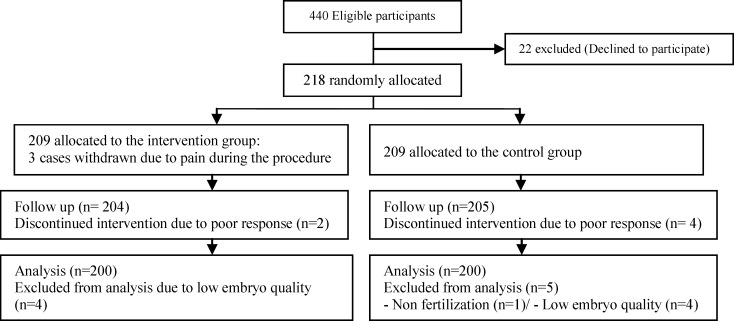
Study flow chart

This prospective study included initially 440 eligible participants. 22 patients refused to take part in study. The remaining 418 participants were allocated in 2 groups. 3 patients were excluded from intervention group as they experienced severe pain during procedure and endometrial scratch was discontinued. Apart from these 3 patients, we had no other patients reported moderate or severe pain during the procedure. During ovarian stimulation, 6 more patients were excluded due to poor response. Nine patients were excluded from analysis due to low embryo quality. None of the patients reported infection or heavy bleeding after the procedure. There was no disturbance in the menstrual cycle other than minimal vaginal spotting for a few days after the procedure.Finally, 200 patients were in each group (Figure 1). 

The baseline characteristics of participants are shown in table I. There was no significant difference between 2 groups regarding chronological age, BMI, duration and causes of infertility, AFC, and baseline hormonal profile. The details of ovarian stimulation are shown in table II. There was no significant difference between 2 groups as regards down-regulation protocol used, total dose and duration of gonadotropin stimulation, number of preovulatory follicles, number of retrieved oocytes, fertilization and cleavage rates, and number of grade A transferred embryos.

**Primary outcome measures**

Implantation rate and live birth rate were significantly higher in intervention compared with control group respectively (22.4% vs. 18.7%, p=0.02, and 67% vs. 28%, p=0.03),.

**Secondary outcome measures**

Clinical pregnancy rate was significantly higher in intervention compared with control group (73.5% vs. 35.5%, respectively, p<0.01). There were 25 cases of multiple pregnancies in intervention group and 8 cases in control group. 

This difference was statistically significant (p<0.001). Miscarriage rate per clinical pregnancy was significantly lower in intervention group (p<0.001). There was one case of tubal ectopic pregnancy in control group. 

**Table I:** Baseline characteristics of the study population

		**Intervention group**	**Control group**	**p-value**
Age (years)		31.4 ± 0.7	30 ± 0.7	0.8
BMI (Kg/m3)		28.9 ± 0.7	27.8 ± 0.4	0.2
Basal FSH (IU/L)		7.9 ± 0.2	7.2 ± 0.3	0.06
Basal LH (IU/L)		7.2 ± 0.3	7.7 ± 0.5	0.4
Basal AMH (ng/ml)		2.3 ± 0.3	2.9 ± 0.3	0.9
AFC		13.8 ± 1.1	16.9 ± 1.1	0.5
Duration of infertility (years)		12 ± 1.3	8.5 ± 0.7	0.05
Cause of infertility				0.7
	Male factor	74 (37%)	75 (37.5%)	
	Tubal factor	40 (20%)	42 (21%)	
	Endometriosis	16 (8%)	17 (8.5%)	
	Unexplained	52 (26%)	53(27.5 %)	
	Anovulation	8 (4%)	7 (3.5%)	
	Combined	10 (5%)	6 (3%)	

Data are presented as mean ± SEM or number (%). (n=200)

**Table II:** Characteristics of ovarian stimulation in both groups

**Down regulation protocol**	**Intervention group**	**Control group**	**p-value**
Long protocol (%)	144 (77%)	146 (78%)	0.9
Short protocol (%)	51 (25.5%)	50 (25%)	
Antagonist (%)	5 (2.5%)	4 (2%)	
Total HMG dose (IU)	2865.4 ± 137	2741.3 ± 184.4	0.9
Duration of stimulation (days)	12.3 ± 0.8	12.1 ± 0.9	0.9
Pre-ovulatory follicle (n).	13.1 ± 2.6	13.3 ± 2.5	0.9
Retrieved oocytes (n)	11.5 ± 1.6	11.6 ± 1.6	0.8
Mature oocytes (n)	10.9 ± 0.7	11 ± 0.8	0.5
Total no. of embryos	8.9 ± 1.2	8.8 ± 1.4	0.6
Embryo transferred (n)	3.2 ± 0.5	3.1 ± 0.6	0.5
Grade A embryo transferred (n)	2.7± 0.4	2.8 ± 0.4	0.6
Fertilization rate (%)	81.4 ± 2.5	75.7 ± 4.6	0.57
Cleavage rate (%)	94.5±0.6	94.7±0.5	0.6

Data are presented as mean ± SEM or percentage (%). (n=200)

**Table III:** The outcome measures in both groups

	**Intervention group**	**Control group**	**p-value**
Implantation rate (%)	22.4%	18.7%	0.02
Clinical pregnancy rate (%)	73.5 %	35.5 %	<0.001
Live birth rate (%)	67 %	28 %	0.03
Miscarriage rate (%)	13/147 (4.8%)	14/71 (19.7%)	<0.001
Multiple pregnancy rate (%)	25/147 (17%)	8/71 (11.3%)	<0.001

Data are presented as mean ± SEM or number (%). (n=200)

## Discussion

Implantation failure remains the main obstacle to IVF/ICSI success. Many trails had been done to improve implantation. One of most promising methods is to induce endometrial injury the luteal phase of the proceeding cycle preceding the stimulation cycle. Although the underlying mechanism of how endometrial injury improves endometrial receptivity remains unclear, inflammatory background is highly suggested. Most of available studies focused on patients with RIF. In our study, it was tried to evaluate influence of endometrial injury on implantation rate, clinical pregnancy rate and live birth rate in patients with first cycle IVF/ICSI. It was also studied effect of endometrial injury on rate of miscarriage per clinical pregnancy and multiple pregnancy rates.

In current study, hysteroscopy was done in a separate setting in follicular phase of preceding cycle as that allowed better evaluation of cavity and exclusion of patients with intra-uterine pathology. In a recent study, hysteroscopy and endometrial biopsy were done at same time in the luteal phase (6). Minor endometrial pathology might be obscured with the thick endometrium in the luteal phase, so performing hysteroscopy was not feasible at this time.. In our study, single endometrial injury with pipelle biopsy catheter was shown to improve significantly the implantation rate, clinical pregnancy rate and live birth rate in patients with first cycle IVF/ICS. There was also a significant reduction in the miscarriage rate per clinical pregnancy. However, significant increase in multiple pregnancy rates was noted.

To our knowledge, there was only one trial included women submitted to their first IVF/ICSI cycle (7). However, in that study, endometrial injury was done at time of ovum pick up and that was associated with significant reduction in clinical and ongoing pregnancy rates. That was most properly due to disruption of growing endometrium at this critical time of IVF/ICSI cycle. Previous studies evaluated endometrial injury included either patients with previous implantation failure or included women, regardless the number of previous IVF attempts (6, 8-12). Except for the study done by Karmizade *et al *all other studies reported improvement in IVF/ICSI outcome with endometrial injury (7).

There is no consensus regarding optimal number of endometrial injuries. In our study, we performed the endometrial injury once between day 21^st^ and to 24^th^ of preceding cycle. In some studies, endometrial injury was performed once like our study (6, 7, 9, 11). Endometrial injury was done twice in other studies (3, 10). There is also no consensus regarding best time to do endometrial injury. There is some suggestion that endometrial injury performed in luteal phase is likely to induce more decidualization. Further studies are needed to compare the effect of single vs. repeated endometrial injuries on IVF/ICSI outcome and to determine the best time of cycle to induce endometrial injury.

In this study, it was found that endometrial injury is associated with significant reduction in miscarriage rate per clinical pregnancy (4.8% vs. 19.7% in intervention and control group respectively p<0.001). A lower miscarriage rate with endometrial injury was reported in one trial (11). However, that was insignificant. It was believed that lower miscarriage rate could be another advantage of endometrial injury. However, it needs to be clarified in larger study. It was also reported significant increase in multiple pregnancy rates in endometrial injury group; another issue needs to be addressed in further well-controlled trials. 

A recent meta-analysis including 901 patients from 8 studies concluded that endometrial injury performed before IVF treatment cycle was associated with significant improvement in outcome and there is a need for well-conducted randomized study to confirm those findings (12). Similar conclusions were obtained by a systematic review done on 2062 patients from 7 studies (13). A recent Cochrane review was done on 591 patients from 5 trials reported similar conclusions and raised issue of the need for large studies to address the beneficial effect of endometrial injury prior to IVF/ICSI (14). The results of our study will be helpful in supporting concept of the need for endometrial injury to be done as routine prior to IVF/ICSI treatment cycle not only in cases with previous implantation failure. 

## Conclusion

In conclusion, endometrial injury induced with Pipelle biopsy sampling is a safe and simple outpatient procedure associated with significant improvement in implantation, clinical pregnancy and live birth rate in IVF/ICSI. We recommend the procedure to be done routinely in all cases undergoing IVF/ICSI in the non-transfer cycle.

## Note

***Registration ID in ISRCTN: ISRCTN61316186***
